# Investigating the Impact of Immune-Related Adverse Events, Glucocorticoid Use and Immunotherapy Interruption on Long-Term Survival Outcomes

**DOI:** 10.3390/cancers13102365

**Published:** 2021-05-14

**Authors:** Charline Lafayolle de la Bruyère, Pierre-Jean Souquet, Stéphane Dalle, Pauline Corbaux, Amélie Boespflug, Michaël Duruisseaux, Lize Kiakouama-Maleka, Thibaut Reverdy, Madeleine Maugeais, Gulsum Sahin, Denis Maillet, Julien Péron

**Affiliations:** 1 Cancer Research Center of Lyon, Oncology Department, Lyon Sud Hospital, Hospices Civils de Lyon, 69495 Pierre-Bénite, France; pauline.corbaux@chu-lyon.fr (P.C.); thibaut.reverdy@chu-lyon.fr (T.R.); madeleine.maugeais@chu-lyon.fr (M.M.); denis.maillet@chu-lyon.fr (D.M.); julien.peron@chu-lyon.fr (J.P.); 2 Université Claude Bernard Lyon 1, 69100 Villeurbanne, France; pierre-jean.souquet@chu-lyon.fr (P.-J.S.); stephane.dalle@chu-lyon.fr (S.D.); amelie.boespflug@chu-lyon.fr (A.B.); michael.duruisseaux@chu-lyon.fr (M.D.); lize.kiakouama-maleka@chu-lyon.fr (L.K.-M.); 3 ImmuCare (Immunology Cancer Research) Institut de Cancérologie des Hospices Civils de Lyon, 69229 Lyon, France; gulsum.sahin@chu-lyon.fr; 4 Cancer Research Center of Lyon, Department of Respiratory Medicine, Lyon Sud Hospital, Hospices Civils de Lyon, 69495 Pierre-Bénite, France; 5 Cancer Research Center of Lyon, Dermatology Department, Lyon Sud Hospital, Hospices Civils de Lyon, 69495 Pierre-Bénite, France; 6 Department of Respiratory Medicine, Groupement Hospitalier Est, Hôpital Louis-Pradel, Hospices Civils de Lyon, 69500 Bron, France; 7 Department of Respiratory Medicine, Croix-Rousse Hospital, Hospices Civils de Lyon, 69004 Lyon, France; 8 CNRS, UMR 5558, Laboratoire de Biométrie et Biologie Évolutive, Équipe Biostatistique-Santé, F-69100 Villeurbanne, France

**Keywords:** immune-related adverse events, glucocorticoid use, ICI interruption, irAEs management

## Abstract

**Simple Summary:**

Immunotherapy has modified our clinical practices for the treatment of various solid cancers. Many studies have been done but it remains unclear whether adverse events induced by immunotherapy and the corticoids used for their management could impact long-term outcomes in patients treated by immunotherapy. A data collection of 828 patients was made to assess the impact of adverse events, immunotherapy interruption and the use of corticoids in long-term outcomes. In this cohort, we did not find any association between adverse events and survival outcomes. However, corticoid use was associated with a significantly shorter time before disease progression. Immunotherapy interruption was associated with a significantly shorter time before progression and survival. The impact of severe adverse events related to immunotherapy reported in other studies might be explained by their management. The use of corticoids must be careful, and resuming immunotherapy after adverse events may be important for long-term prognosis and should be considered as often as possible.

**Abstract:**

It remains unclear whether immune-related adverse events (irAEs) and glucocorticoid use could impact long-term outcomes in patients treated for solid tumors with immune checkpoint inhibitors (ICI). All patients treated with a single-agent ICI for any advanced cancer were included in this retrospective unicentric study. The objectives were to assess the impact of grade ≥3 irAEs, glucocorticoid use and the interruption of immunotherapy on progression-free survival (PFS) and overall survival (OS). In this 828-patient cohort, the first occurrence of grade ≥3 irAEs had no significant impact on PFS or OS. Glucocorticoid administration for the irAEs was associated with a significantly shorter PFS (adjusted HR 3.0; *p* = 0.00040) and a trend toward shorter OS. ICI interruption was associated with a significantly shorter PFS (adjusted HR 3.5; *p* < 0.00043) and shorter OS (HR 4.5; *p* = 0.0027). Glucocorticoid administration and ICI interruption were correlated. In our population of patients treated with single agent ICI, grade ≥3 irAEs did not impact long-term outcomes. However, the need for glucocorticoids and the interruption of immunotherapy resulted in poorer long-term outcomes. The impact of grade ≥3 irAEs reported in other studies might then be explained by the management of the irAEs.

## 1. Introduction

Immune checkpoint inhibitors (ICI) have successfully modified clinical practice for treatment of several solid malignancies [1]. Metastatic lung cancer, melanoma and renal cancer are good examples, as the routine therapeutic strategies now include ICI, such as anti-cytotoxic T-lymphocyte antigen4 (CTLA-4), or anti-programmed cell death 1 (PD1) or its ligand (PDL1) [2,3,4,5,6,7,8,9,10].

By increasing immune activity, ICI can also generate dysimmune toxicities, called immune-related adverse events (irAEs). IrAEs mainly involve the skin, endocrine glands (thyroid, pancreas, hypophysis), liver, lungs and gastro-intestinal tract, but can affect every organ [11,12]. With single agents, these events are frequent but mostly of low grade (symptomatic treatment allowing ICI pursuit) whereas grade ≥3 irAEs (requiring hospitalization due to interference with the basic activities of daily life) occur among less than 15% patients treated with an anti-PD(L)-1 and approximately 40% of patients treated with an anti-CTLA-4 [13].

Some authors have suggested that irAEs could be a surrogate of immune activation and so correlated with ICI efficacy. This correlation was demonstrated with vitiligo-like depigmentation for patients treated with immunotherapy for melanoma [14,15] but results are conflicting for all other cases [16,17,18,19,20,21,22].

Glucocorticoids have been associated with a decreased efficacy of ICI through their immunosuppressive effect but with conflicting results. Some authors advise avoiding the use of >10 mg of prednisone in association with immunotherapy [23], but a recent meta-analysis of 27 studies did not find any association between glucocorticoid use and ICI efficacy [24].

A large real-life cohort of patients with various malignancies receiving ICI was used to assess the impact of grade ≥3 irAEs on long-term oncological outcomes (PFS and OS). As grade ≥3 irAEs can lead to glucocorticoid introduction and ICI interruption, we also investigated the impact of such practices on oncological outcomes.

## 2. Materials and Methods

### 2.1. Patients

All patients with locally advanced or metastatic solid tumor, such as melanoma, non-small cell lung cancer or urologic cancer who started a treatment with ICIs as single agent (PD(L)-1 inhibitors or CTLA-4 inhibitors), between January 2007 and December 2018 in one of the three subsites of the Lyon University Hospital, France, were included. This study has been approved by the Ethical Review Board of the Hospices Civils de Lyon (n*19–21).

### 2.2. Data Collection

Data collection was performed retrospectively using a standardized data collection form.

Patient and disease characteristics at the start of ICI were extracted: age, gender, Eastern Cooperative Oncology Group performance status, primary tumor (melanoma, lung cancer or urologic cancer (bladder or renal cancer)), number of metastatic sites and presence of brain, bone or visceral metastases.

Adverse events were categorized as irAEs when at least possibly attributed to the ICI, based on the judgement of the treating physicians. The grading of irAE severity was based on the Common Terminology Criteria for Adverse Events, version 4.0. IrAEs included pneumonitis, colitis, thyroiditis, dermatitis, colitis, hepatitis or any adverse events at least possibly related to an activation of the immune system according to the treating physician. Only grade 3 and more irAEs were considered in this study.

Clinical follow-up was scheduled at each ICI administration. Imaging follow-up was scheduled every 2 or 3 months according to local clinical practice. The date of disease progression was assessed by the treating physician based on clinical examination and imaging.

The glucocorticoid starting dose was collected according to the weight of patient (mg/kg). Only glucocorticoid use related to irAEs was collected, and only by systemic way (local application was not considered). Dates of ICIs interruption and its possible resumption were collected.

### 2.3. Statistical Analysis

The characteristics of patients were described according to the type of ICI administered. The frequency of irAEs was described according to the type of ICI and primary tumor. Fischer exact test was used to compare binary or qualitative variables including the rates of irAEs according to age. Mann−Whitney test was used to compare quantitative variables.

Overall survival (OS) was defined as the time from treatment initiation to death from any cause. Progression free survival (PFS) was defined as the time from treatment initiation to progressive disease or death from any cause, whichever came first.

The time since treatment start of irAEs was described by irAEs severity grade. Only the first occurrence per grade of irAE was described for each patient. Only ICI interruptions related to irAEs were considered in the definition.

As irAEs might happen late during the follow-up and progression event or death might happen early, an immortal time bias might occur as patients responding to ICI will receive the ICI and then be exposed to irAEs for a longer period of time. The first occurrence of an irAE was then included in Cox models, as a time-varying covariate. In the time-varying Cox models, group membership ass changed from “no irAE” to “irAE” at the time the irAE occurred. The hazard ratios (HR) estimated through time-varying Cox models took into account the immortal time bias.

As the impact of irAEs on PFS and OS was expected to be modified by an introduction of glucocorticoids or by the interruption of the ICI, this was assessed in the population of patients with at least one grade ≥3 irAE. The impact of glucocorticoid initiation used for the treatment of an irAE and ICI interruption on PFS and OS was also studied in time-varying Cox models. Multivariate Cox models were conducted to adjust for potential cofounders (performance status, age, treatment line, presence of visceral metastases, primary tumor and ICI type).

All analyses were performed using R statistical software (R Foundation for Statistical Computing). Database follow-up was locked in June 2020. Data were rarely missing, and no data imputation was necessary through the analyses.

## 3. Results

### 3.1. Patients, Disease and Treatment Characteristics

We included 864 lines of treatment administred to 828 patients. Thus, 36 patients received two separate lines of immunotherapy, all treated for a melanoma. Patients baseline characteristics and comparison between patients with and without irAEs are summarized in Table 1. Median age was 66 years (95% CI 58–73). Of the treatment lines, 780 (90%) were anti-PD(L)-1, whereas 84 (10%) were anti-CTLA-4. All 84 single agent anti-CTLA-4 were prescribed for melanoma patients, with an ipilimumab dose of 3 mg/kg.

Most patients (555 of 828, 67%) had non-small cell lung cancer (NSCLC) and all of them received only one ICI line.

The 194 melanoma patients (23%) received a total of 230 ICI lines. Lastly, 79 patients (10%) had urological cancer (clear cell renal carcinoma or urothelial carcinoma) and received only one ICI line. Furthermore, 224 patients (26%) received ICI as third line or more.

Median follow-up after first administration was 42.3 months (95% CI 40.6-NR) among patients treated with a CTLA4-inhibitor and 15.9 months (95% CI 14.7–17.3) among those treated with a PD(L)-1 inhibitor.

Among the 11 patients included in our study with an immuno-induced interstitial lung disease, 3 had radiotherapy before immunotherapy introduction.

### 3.2. Immune-Related Adverse Events Characteristics and Glucocorticoid Use

Overall, grade ≥3 irAEs occurred in 78 patients (9 %). They were more frequent with anti-CTLA-4 (24%), compared with anti-PD(L)-1 (7 %) (Table 2). Grade 4 irAEs occurred in 13 patients including 4 in the anti-CTLA-4 group and 9 in the anti-PD(L)-1 group. Only one grade 5 irAE was reported, in a patient treated by an anti-PD(L)-1 for a urological cancer.

Median time to the first grade ≥3 irAE was 1.2 months (95% CI 0.5–2.9). Most grade ≥3 irAEs occurred into the first 3 months, and only 13% occurred after 6 months.

Immune colitis was the most frequent grade ≥3 irAE, followed by immune hepatitis, immune rash and hypophysitis.

Six cases of immune pneumopathy, four cases of immune cardiac AE (myocarditis mostly) and 20 other grade ≥3 irAEs (Guillain−Barre syndrome, diabetes, pancreatitis, arthritis, etc.) were reported.

Among patients treated with anti-PD(L)-1, the incidence of irAEs was relatively similar among patients with melanoma compared with patients with lung or urologic cancer (9% versus 7% versus 6%, respectively, *p* = 0.74).

Among patients with grade ≥3 irAEs, 65% of patients with anti-CTLA-4 and 55% of patients with anti-PD(L)-1 received glucocorticoids to manage irAEs (Table 3).

Patients with anti-CTLA-4 received higher doses of glucocorticoids (1 mg/kg for 38% of patients, 2 mg/kg and more for 61% of patients) than patients treated by anti-PD(L)-1 (0.5 mg/kg for 22% of patients, 1mg/kg for 62% of patients and 2 mg/kg and more for 16% of patients). Another immunosuppressive treatment was necessary in 10% of cases overall (mostly TNF-alpha inhibitors for colitis).

Treatment was interrupted after grade ≥3 irAEs in 70% of patients treated with an anti-CTLA-4 and 86% of patients treated with an anti-PD(L)-1. Table 3 also describes management of irAEs according to their type.

The proportion of patients receiving glucocorticoids and the proportion of patients with an ICI discontinuation for the management of a grade ≥3 irAE were stable over time (before 2016 vs. 2016–2017 vs. after 2017). (Table 4)

Reintroduction was done in 30% of cases in the two groups, and a new irAE appeared in 50% of patients treated with an anti-CTLA-4 and 30% of patients treated with an anti-PD(L)-1 (Table 3). A multidisciplinary meeting was set up in our institution to discuss these cases and particularly the reintroduction of ICI.

Overall, 258 patients presented only one grade ≥3 irAE (about 31% of our cohort), 112 patients presented 2 grade ≥3 irAEs (13%), 28 patients presented 3 grade ≥3 irAEs (3%) and 7 patients presented 4 grade ≥3 irAEs (1%).

### 3.3. Association between Immune-Related Adverse Events and Long-Term Outcomes

In the overall population, the occurrence of a grade ≥3irAE had no statistically significant effect on PFS (HR 0.94; 95%CI 0.7–1.26; *p* = 0.70) or OS (HR 0.82; 95% CI 0.6–1.12; *p* = 0.21).

**Figure 1:**
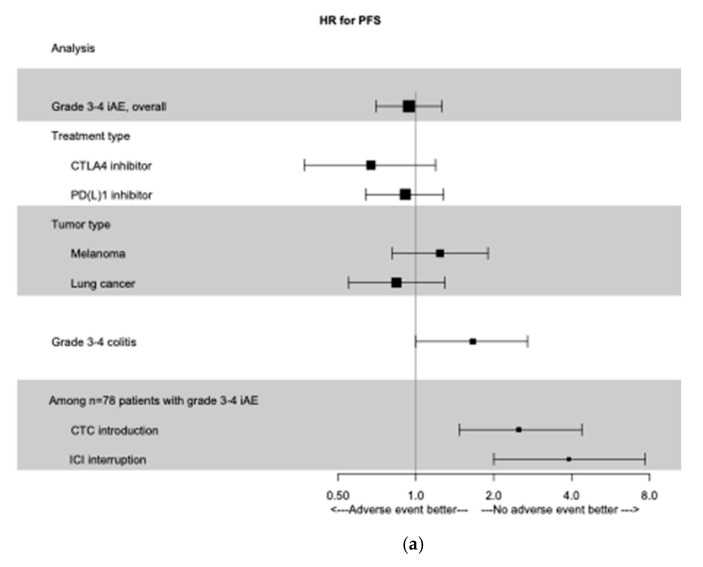

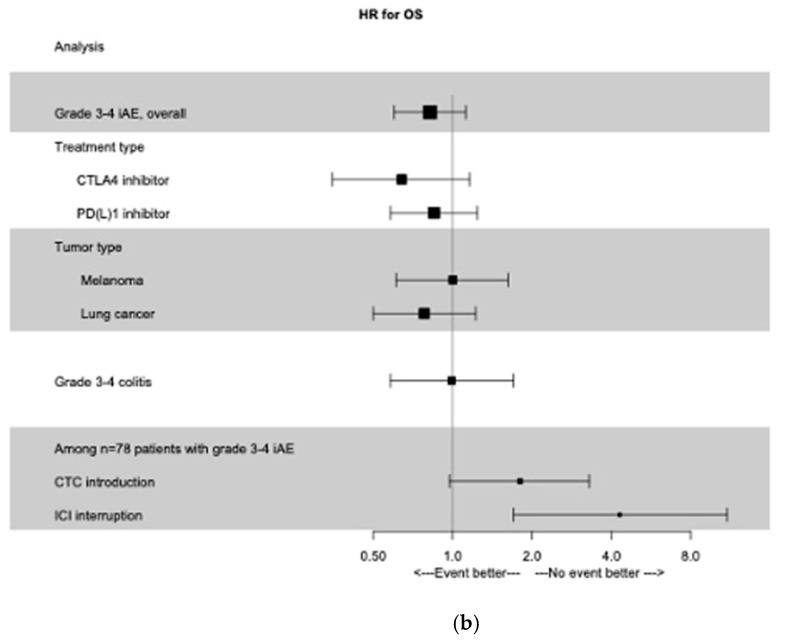
Time-dependent Forest Plot analysis. (**a**) Forest Plot of PFS according to grade 3−4 irAEs, treatment type, primary tumor type, and management of irAEs. (**b**) Forest Plot of OS according to grade 3−4 irAEs, treatment type, primary tumor type, and management of irAEs. irAEs, immune-related adverse events; PFS, progression free survival; OS, overall survival; CTC: glucocorticoids; ICI: immune check-point inhibitors.

This lack of association was consistent in subgroups of patients treated with an anti-CTLA-4 (HR for PFS 0.67; 95% CI 0.37–1.19; HR for OS 0.64; 95% CI 0.35–1.16) or an anti-PD(L)-1 (HR for PFS 0.91; 95% CI 0.64–1.28 and HR for OS 0.85; 95% CI 0.58–1.24). Results were consistent in subgroups of patients treated for melanoma or pulmonary cancer. All these results are summed up in Figure 1.

### 3.4. Association between Glucocorticoid Use and Long-Term Outcomes

Among patients with grade ≥3 irAEs, those receiving glucocorticoids had a shorter PFS (unadjusted HR for PFS 2.5; 95% CI 1.5–4.4; *p* = 0.00080). A similar negative effect was observed for OS but was not statistically significant (unadjusted HR for OS 1.80; 95% CI 1–3.3; *p* = 0.061). Results were consistent in the multivariate analysis (adjusted HR for PFS 3.0; 95% CI 1.6–5.4; *p* = 0.00040 and adjusted HR for OS 1.8; 95% CI 0.9–3.4; *p* = 0.083). However, among the whole cohort of patients, introduction of glucocorticoids for grade ≥3 irAEs did not impact the PFS (adjusted HR 1.3; 95% CI 0.91–2.0; *p* = 0.14) or OS (adjusted HR 0.99; 95% CI 0.66–1.5; *p* = 0.96).

### 3.5. Association between Immunotherapy Interruption and Long-Term Outcomes

Among patients with grade ≥3 irAEs, PFS was significantly shorter for those who stopped immunotherapy (analysis with time dependant covariate, unadjusted HR 3.9; 95% CI 2.0–7.7; *p* < 0.0001). OS was also significantly shorter for these patients (unadjusted HR 4.3; 95% CI 1.7–11.0; *p* = 0.0024). Results were consistent in the multivariate analysis (adjusted HR for PFS 3.5; 95% CI 1.7–6.0; *p* = 0.00043 and adjusted HR for OS 4.5; 95% CI 1.7–12.1; *p* = 0.0027) (Figure 1*).*

Among the whole cohort of patients, immunotherapy interruption for grade ≥3 irAEs did not impact the PFS (adjusted HR 1.3; 95% CI 0.92–1.7; *p* = 0.15) or OS (adjusted HR 1.0; 95% CI 0.74–1.43; *p* = 0.87).

We include in the Supplementary Materials modelized survival curves, taking into account the immortal time bias.

Finally, we studied the correlation between the use of glucocorticoids and the interruption of immunotherapy in patients with at least one grade ≥3 irAE. Some 66% of patients who needed glucocorticoids also stopped immunotherapy whereas 79% of patients who did not need glucocorticoids continued immunotherapy (Table 5a).

There was no correlation between the type of irAE and the ICI interruption (Table 5b), but a correlation was found between the type of irAEs and the use of glucocorticoids, with a major use for colitis, myositis, pneumonitis and hepatitis but not for diabetes or hypophysitis (Table 5c).

## 4. Discussion

In this large cohort of patients, we did not find any significant association between grade ≥3 irAEs and long-term outcomes. Results were consistent in the subgroups of patients treated with anti-CTLA-4 or anti-PD(L)-1, and in subgroups of patients treated for melanoma or pulmonary cancer.

Among patients with grade ≥3 irAEs, those receiving glucocorticoids had a shorter PFS but the association was not statistically significant for OS. In the same way, patients who stopped immunotherapy had a significant shorter PFS and OS.

There was a strong correlation between the use of glucocorticoids and interruption of immunotherapy.

These results are in conflict with an earlier analysis performed on the same cohort, also conducted by Hospices Civils de Lyon’s medical team [25]. This previous analysis, performed on a smaller cohort and with shorter follow-up, found a positive association between grade ≥2 irAEs and long-term outcomes with an HR for PFS of 0.63 (95% CI 0.50–0.81; *p* = 0.00022) and an HR for OS of 0.57, (95% CI 0.43–0.74; *p* < 0.0001).

Grade 2 irAEs were included in this previous analysis. Thyroiditis and dermatologic AEs, both AEs frequently of grade 2, were significantly associated with better outcomes, while colitis and hepatitis were not associated with a better prognosis. The limitation of the current analysis to grade ≥3 irAEs, might partly explain this difference. The impact of irAEs on long-term outcomes may also have varied over time, as a consequence of a modification in the clinical management of irAEs.

Many other studies have shown a positive association between irAEs and long-term outcomes, but the analyses were sometimes potentially impacted by an immortal time bias, as patients had to be alive and progression-free at the time of their first irAE to be classified in the irAE group.

Nevertheless, a recent meta-analysis published by Haratani et al. showed this correlation, respecting the immortal time bias [26]. The same data are available for urothelial carcinoma [27], melanoma [28] or non-small cell lung cancer [29] independently.

Eggermont et al. [20] in a secondary analysis of a randomized clinical trial assessing pembrolizumab versus placebo in melanoma adjuvant situation, found a positive association between irAEs and long-term outcomes, all grades included, taking into account the bias of immortality (RFS HR = 0.37 for patients with irAEs, HR = 0.62 for others; *p* < 0.03).

An association between cutaneous toxicities and better long-term outcomes has been reported [21,22]. Similar self-antigens were found in skin and tumor tissue in lung cancers, but also in melanoma [30] which might explain these cross reactions. A recent study has shown the same link between acute interstitial nephritis and immunotherapy response in patients treated for a renal cell carcinoma, potentially also explained by antigenic overlap [31].

Among our 78 patients with grade ≥3 irAEs, glucocorticoid use was associated with a shorter PFS. Management of irAEs is complex, as clinicians are looking for a balance in the immune activation between efficacy and toxicity. Major clinical guidelines (ASCO, American Society of Clinical Oncology, and ESMO, European Society for Medical Oncology) have proposed standardized procedures to manage irAEs, involving a rapid use to glucocorticoids [32,33]. They can be used from grade ≥2 irAEs (0.5–1 mg/kg and suspension of ICI up to a return to grade I or less following ASCO recommendations). From grade 3, reintroduction is discussed, and caution is advised especially in patients with early onset irAEs. Yet, different studies have shown a negative impact of glucocorticoids from 10 mg per day [23,34]. A recent study described a lower immunotherapy efficacy according to the glucocorticoids dose, duration, and the delay between the immunotherapy start and glucocorticoids initiation [35].

Douglas et al. investigated the association between glucocorticoid dose and long-term outcomes among melanoma patients with hypophysitis induced by ipilimumab [36]. Patients who received low-dose steroids had better clinical outcomes in terms of time to treatment failure (HR 0.28; *p* = 0.01) and OS (HR 0.24; *p* = 0.002), hypophysitis being one grade ≥3 irAE that does not require high doses of glucocorticoids for its treatment.

Eggermont et al. also reported a lower efficacy of pembrolizumab in patients treated for 30 days or more with glucocorticoids (HR = 0.50 versus 0.34 for other patients) [20].

A meta-analysis including more than 4000 patients with 16 studies, showed an increased mortality for patients treated with glucocorticoids among all patients treated with ICI, especially for patients for whom indication was supportive care (HR 2.5; 95% CI 1.41–4.43; *p* < 0.01) or brain metastases (HR 1.51; 95% CI 1.22–1.87; *p* < 0.01) [37]. In this meta-analysis, outcomes were not significantly lower among patients whom glucocorticoids indication was management of irAEs. However, this conclusion might be subject to immortal time bias.

The last interesting result of our study was the association between interruption of immunotherapy and long-term outcomes. Given the high correlation between ICI interruption and use of glucocorticoids for the management of an irAE, we could not distinguish which of the two factors had a causal negative impact on long-term outcomes.

A recent prospective monocentric study included all melanoma patients discussed at a tumor board, and aimed to analyze disease outcomes with resumption of immunotherapy as compared to nonresumption of immunotherapy after a severe irAE [38]. This small study (26 patients) suggested that the ability to resume ICI after an irAE was associated with better prognosis.

Our study did not include enough patients to study the impact of temporary or permanent ICI interruption, and only 30% of our patients resumed immunotherapy.

In contrast, an update of the phase 3 study evaluating nivolumab + ipilimumab versus sunitinib in clear cell renal carcinoma showed no negative impact on OS of irAEs incidence but also of therapy discontinuation due to treatment-related irAEs [39]. The difference with our study may be that few of our patients were treated with ipilimumab with therefore a majority of treatment with anti-PD(L)-1.

A significant negative impact of grade 3 and 4 colitis on PFS was found in our study using analysis with time dependant covariate (HR 1.66; 95% CI 1.0–2.7; *p* = 0.047). No significant difference was found for OS. This result seems consistent with previously discussed findings, since colitis often requires a high dose of glucocorticoids and immunotherapy is often permanently withdrawn.

Furthermore, the use of glucocorticoids seems to be deleterious on long term-outcomes, but results are inconsistent depending on the dose, delay and reason for their use. The link with immunotherapy interruption is major and does not allow us to conclude on the cause of this negative impact. Anyway, resumption must always be cautious because about 25% of patients will have a new irAE and 25% will have a recurrence of their initial irAE [40]. There are no studies evaluating the relationship between the use of other immunosuppressive treatments and long term-outcomes, but they are not used in the first intervention in the management of irAEs, due to their own adverse events [41,42].

Finally, the question is raised about the need for prolonged treatment with immunotherapy. One of the efficacy hypotheses was the reactivation of the immune system as a result of the first injections and the release of tumoral neo antigens. Robert et al. assessed the impact of the reintroduction of ICI in the case of progression after discontinuation for patients with nonprogressive disease after one year of treatment with durvalumab for various solid tumors [43]. Among 70 re-treated patients, 60% patients had a stable disease and 11.4% had a partial response, highlighting the value of a long-term treatment by immunotherapy and not just a prolonged initial response, and so a negative impact of ICI interruption.

Our study has some limitations. Its retrospective nature makes it at risk of misclassification bias, but it is notable that the amount of missing data was very low, and it included a large number of patients. Only grade 3 and 4 irAEs were included in the analysis, as grade 2 irAEs were suspected to be less exhaustively described in medical files used retrospectively to collect data. The relation with the ICI of these severe adverse events is also expected to be easier to establish by clinicians.

The study included patients with various cancer types, as the relationship between irAEs, irAEs management, and ICI efficacy was hypothesized to be similar according to tumor types. However, we cannot exclude that some mechanisms might be tumor specific. For example, the link between vitiligo occurrence and ICI efficacy might be specific to melanoma patients.

Moreover, given the restrospective nature of this study, the decision to introduce glucocorticoids and the decision to interrupt ICI was at the discretion of the treating physician. It is possible that these decisions have been influenced by the severity and type of the irAE but also by the perceived efficacy of the treatment. This might have introduced a bias in the estimation of the impact of glucocorticoids and ICI interruption on long-term oncological outcomes.

We only focused on patients treated with ICI as single agent, but more and more, combinations of ICI with other treatments are now used in clinical practice. The relation between irAEs and outcomes will be harder to explore in patients receiving such combinations.

## 5. Conclusions

Our findings suggest no strong association between grade ≥3 irAEs and long-term outcomes in patients treated with ICI single agent for various solid cancers. Many other studies found an association between irAEs and long-term outcomes but all irAEs grades were included and immortal time bias was not necessarily tackled. However, an association was found between the use of glucocorticoids and the interruption of immunotherapy and long-term outcomes.

Given the strong association between glucocorticoid use and ICI interruption, we cannot establish a causal link between one of these two elements and long-term outcomes but resuming immunotherapy after irAEs may be important for long-term outcomes and should therefore be considered as often as possible. Glucocorticoid use must be careful, and clinicians must have in mind to use the minimal efficient dose of glucocorticoid in order to maintain a balance between the benefit and toxicity of ICIs.

## Figures and Tables

**Table 1:** Patients characteristics, AE = Adverse Event; BMI = Body mass index; PS = Performans Status.

Variable	All Treatment Types (n = 864)				CTLA-4 Inhibitor (n = 84)				PD(L)1 Inhibitor (n = 780)			
	All Patients	Patients without Grade ≥ 3 Immune Related AE (n = 786)	Grade ≥ 3 Immune Related AE (n = 78)	*p*	All Patients	Patients without Grade ≥ 3 Immune Related AE (n = 64)	Grade ≥ 3 Immune Related AE (n = 20)	*p*	All Patients	Patients without Grade ≥ 3 Immune Related AE (n = 722)	Grade ≥ 3 Immune Related AE (n = 58)	*p*
Age, years, median (25th–75th) NA = 0	66 (58–73)	66 (57–73)	65 (56–72)	0.41	63 (49–70)	63 (50–73)	61 (49–69)	0.58	66 (58–73)	66 (58–73)	65 (59–75)	0.97
Gender (%) NA = 0				0.12				0.41				0.047
Male	603 (70%)	555 (71%)	48 (62%)		50 (60%)	36 (56%)	14 (70%)		553 (71%)	519 (72%)	34 (59%)	
Female	261 (30%)	231 (29%)	30 (38%)		34 (40%)	28 (44%)	6 (30%)		227 (29%)	203 (28%)	24 (41%)	
PS ≥ 2 (%) NA = 34	207 (25%)	193 (26%)	14 (19%)	0.21	15 (18%)	12 (19%)	3 (15%)	1.0	192 (26%)	181 (26%)	11 (20%)	0.34
BMI (%) NA = 8				0.77				0.57				
<18	63 (7%)	56 (7%)	7 (9%)		3 (4%)	3 (5%)	0 (0%)		60 (8%)	53 (7%)	7 (12%)	
18–30	684 (80%)	623 (80%)	61 (79%)		68 (81%)	50 (78%)	18 (90%)		616 (79%)	573 (80%)	43 (75%)	
>30	109 (13%)	100 (13%)	9 (12%)		13 (16%)	11 (17%)	2 (10%)		96 (12%)	89 (12%)	7 (12%)	0.36
Primary tumor				<0.0065				1				0.74
Lung cancer	555 (64%)	515 (65%)	40 (51%)		0 (0%)	0 (0%)	0 (0%)		555 (71%)	515 (71%)	40 (69%)	
Melanoma	230 (27%)	197 (25%)	33 (42%)		84 (100%)	64 (100%)	20 (100%)		146 (19%)	133 (18%)	13 (22%)	
Urologic cancer	79 (9%)	74 (9%)	5 (6%)		0 (0%)	0 (0%)	0 (0%)		79 (10%)	74 (10%)	5 (9%)	
≥ 3 metastatic sites (%) NA = 0	306 (35%)	276 (35%)	30 (39%)	0.62	44 (52%)	32 (50%)	12 (60%)	0.46	262 (34%)	244 (34%)	18 (31%)	0.77
Known brain metastases (%) NA = 0	229 (27%)	212 (27%)	17 (22%)	0.39	23 (27%)	18 (28%)	5 (25%)	1	206 (26%)	194 (27%)	12 (21%)	0.38
Bone metastases (%) NA = 0	282 (33%)	256 (33%)	26 (33%)	0.99	14 (17%)	9 (14%)	5 (25%)	0.42	268 (34%)	247 (34%)	21 (36%)	0.87
Visceral metastases (%) NA = 0	646 (75%)	586 (75%)	60 (77%)	0.75	71 (85%)	54 (84%)	17 (85%)	1	575 (74%)	532 (74%)	43 (74%)	1.0
≥3rd line in metastatic setting (%) NA = 7	224 (26%)	207 (27%)	17 (22%)	0.43	16 (19%)	14 (22%)	2 (10%)	0.73	208 (27%)	193 (27%)	15 (26%)	0.97
Smoking habits (%) NA = 41				0.73				0.15				0.51
Active	249 (30%)	224 (30%)	25 (33%)		14 (17%)	11 (18%)	3 (15%)		235 (32%)	213 (31%)	22 (39%)	
Stopped > 1 year	366 (45%)	335 (45%)	31 (40%)		16 (20%)	9 (15%)	7 (35%)		350 (47%)	326 (48%)	24 (42%)	
Never	208 (25%)	187 (25%)	21 (27%)		52 (63%)	42 (68%)	10 (50%)		156 (21%)	145 (21%)	11 (19%)	
Any history of autoimmune disorder (%) NA = 6	85 (10%)	73 (9%)	12 (15%)	0.19	11 (13%)	8 (13%)	3 (15%)	0.72	74 (10%)	65 (9%)	9 (15%)	0.22
Any immune related AE (%) NA = 0	410 (47%)	_	_	_	54 (64%)	_	_	_	356 (46%)	_	_	_

**Table 2:** Description of irAEs. irAEs = immune-related Adverse Events.

Variable	CTLA-4 Inhibitors	PD(L)-1 Inhibitors			
	Melanoma (n = 84)	All Tumor Types (n = 780)	Lung Cancer (n = 555)	Melanoma (n = 146)	Urologic Cancer (n = 79)
Immune AE, grade ≥ 3 (%)	20 (24%)	58 (7%)	40 (7%)	13 (9%)	5 (6%)
Immune colitis, grade ≥ 3 (%)	9 (11%)	15 (2%)	7 (1%)	4 (3%)	4 (5%)
Immune rash, grade ≥ 3 (%)	2 (2%)	9 (1%)	8 (1%)	1 (1%)	0 (0%)
Hypophysitis, grade ≥ 3 (%)	4 (5%)	6 (1%)	2 (0%)	3 (2%)	1 (1%)
Immune hepatitis, grade ≥ 3 (%) NA = 0	6 (7%)	6 (1%)	4 (1%)	2 (1%)	0 (0%)
Immune pneumopathy, grade ≥ 3 (%)	1 (5%)	5 (1%)	8 (1%)	1 (1%)	1 (1%)
Immune cardiac AE, grade ≥ 3 (%)	0 (0%)	4 (1%)	4 (1%)	0 (0%)	0 (0%)
Immune nephritis, grade ≥ 3 (%)	0 (0%)	1 (0%)	0 (0%)	1 (1%)	0 (0%)
Other immune AE, grade ≥ 3 (%)	3 (4%)	17 (2%)	13 (2%)	3 (2%)	1 (1%)

**Table 3:** Management of grade ≥3 irAEs. irAEs = immune-related Adverse Events; ICI = immune check-point inhibitors.

Management	CTLA-4 Inhibitors	PD(L)-1 Inhibitors	Gr ≥ 3 Colitis	Gr ≥ 3 Hypophysitis	Gr ≥ 3 Dermatitis	Gr ≥ 3 Hepatitis
	Melanoma (n = 20)	All Tumors Type (n = 58)	n = 24	n = 10	n = 11	n = 12
Glucocorticoids use, number of patients	13 (65%)	32 (55%)	14 (74%)	4 (40%)	6 (55%)	7 (58%)
Glucocorticoid dose						
≤0.5 μγ/κγ	0 (0%)	7 (22%)	0 (0%)	0 (0%)	1 (17%)	0 (0%)
1 mg/kg	5 (38%)	20 (62%)	10 (71%)	2 (50%)	3 (50%)	3 (43%)
≥2 μγ/κγ	8 (61%)	5 (16%)	4 (29%)	2 (50%)	2 (33%)	4 (57%)
Glucocorticoids attack treatment duration in days, median (25th–75th)	42 (30–58)	47 (29–84)	40 (30–77)	20 (8–33)	39 (35–52)	42 (30–44)
Glucocorticoids maintenance, number of patients	5 (25%)	18 (31%)	6 (25%)	1 (10%)	3 (27%)	4 (33%)
Other immunosuppressive treatment	2 (10%)	6 (10%)	2 (8%)	1 (10%)	2 (18%)	1 (8%)
ICI interruption	14 (70%)	50 (86%)	16 (84%)	5 (50%)	8 (73%)	10 (83%)
ICI reintroduction	4 (29%)	16 (32%)	3 (19%)	3 (60%)	3 (38%)	3 (30%)
Time to reintroduction, in days, median (25th–75th)	36 (31–42)	39 (24–72)	28 (28–48)	45 (37–85)	31 (26–96)	41 (36–45)
New irAE after reintroduction	2 (50%)	7 (32%)	0 (0%)	3 (100%)	0 (0%)	1 (33%)

**Table 4:** Management of irAEs over time. irAEs = immune related Adverse Events; CTC = glucocorticoids; ICI = immune check-point inhibitors.

	<2016, n = 25	2016–2017 , n = 32	>2017, n = 21	*p*
ICI interruption	18 (72%)	30 (94%)	16 (76%)	0.069
CTC use	16 (64%)	17 (53%)	12 (57%)	0.71

**Table 5:** Correlation between the type of irAEs and their management. (a) Correlation between interruption if ICI and introduction of glucocorticoids. (b) Correlation between interruption of ICI and the type of irAEs. (c) Correlation between the type of irAEs and introduction of glucocorticoids. irAEs = immune-related adverse events;. ICI = immune check-point inhibitors; CTC = glucocorticoids.

**(a) *p* value Fisher test = 0.0056**		
	**No CTC**	**CTC**
No interruption	11 (79%)	3 (21%)
Interruption	22 (34%)	42 (66%)
**(b) *p* value Fisher test = 1.0**		
	**Other irAE**	**Colitis, myositis, pneumonitis, hepatitis**
No interruption	10 (71%)	4 (29%)
Interruption	43 (67%)	21 (33%)
**(c) *p* value Fisher test = 0.029**		
	**Other gr3-4 irAE**	**Gr 3-4 Colitis, myositis, pneumonitis, hepatitis**
No CTC	27 (82%)	6 (18%)
CTC	26 (58%)	19 (42%)

## Data Availability

The data presented in this study are available on request from the corresponding author. The data are not publicly available due to privacy.

## Supplementary Materials

The following are available online at https://www.mdpi.com/article/10.3390/cancers13102365/s1, Figure S1: Overall survival (OS) depending on the use of glucocorticoids or not in patients with grade 3 and more immune-related adverse events (irAEs), Figure S2 : Progression free survival (PFS) depending on the use of glucocorticoids or not in patients with grade 3 and more irAEs; Figure S3 : OS depending on the interruption of immune check-point inhibitors or not in patients with grade 3 and more irAEs; Figure S4 : PFS depending on the interruption of immune check-point inhibitors or not in patients with grade 3 and more irAEs.

## References

[1] Velcheti V., Schalper K. (2016). Basic Overview of Current Immunotherapy Approaches in Cancer. Am. Soc. Clin. Oncol. Educ. Book.

[2] Larkin J., Chiarion-Sileni V., Gonzalez R., Grob J.J., Cowey C.L., Lao C.D., Schadendorf D., Dummer R., Smylie M., Rutkowski P. (2015). Combined Nivolumab and Ipilimumab or Monotherapy in Untreated Melanoma. N. Engl. J. Med..

[3] Motzer R.J., Tannir N.M., McDermott D.F., Frontera O.A., Melichar B., Choueiri T.K., Plimack E.R., Barthélémy P., Porta C., George S. (2018). Nivolumab plus Ipilimumab versus Sunitinib in Advanced Renal-Cell Carcinoma. N. Engl. J. Med..

[4] Borghaei H., Paz-Ares L., Horn L., Spigel D.R., Steins M., Ready N.E., Chow L.Q., Vokes E.E., Felip E., Holgado E. (2015). Nivolumab versus Docetaxel in Advanced Nonsquamous Non-Small-Cell Lung Cancer. N. Engl. J. Med..

[5] Bellmunt J., De Wit R., Vaughn D.J., Fradet Y., Lee J.-L., Fong L., Vogelzang N.J., Climent M.A., Petrylak D.P., Choueiri T.K. (2017). Pembrolizumab as Second-Line Therapy for Advanced Urothelial Carcinoma. N. Engl. J. Med..

[6] Rini B.I., Plimack E.R., Stus V., Gafanov R., Hawkins R., Nosov D., Pouliot F., Alekseev B., Melichar B., Vynnychenko I. (2019). Pembrolizumab plus Axitinib versus Sunitinib for Advanced Renal-Cell Carcinoma. N. Engl. J. Med..

[7] Gandhi L., Rodríguez-Abreu D., Gadgeel S., Esteban E., Felip E., De Angelis F., Domine M., Clingan P., Hochmair M.J., Powell S.F. (2018). Pembrolizumab plus Chemotherapy in Metastatic Non-Small-Cell Lung Cancer. N. Engl. J. Med..

[8] Paz-Ares L., Luft A., Vicente D., Tafreshi A., Gümüş M., Mazières J., Hermes B., Şenler F.Ç., Csőszi T., Fülöp A. (2018). Pembrolizumab plus Chemotherapy for Squamous Non-Small-Cell Lung Cancer. N. Engl. J. Med..

[9] Reck M., Rodríguez-Abreu D., Robinson A.G., Hui R., Csőszi T., Fülöp A., Gottfried M., Peled N., Tafreshi A., Cuffe S. (2016). Pembrolizumab versus Chemotherapy for PD-L1–Positive Non–Small-Cell Lung Cancer. N. Engl J. Med..

[10] Herbst R.S., Baas P., Kim D.-W., Felip E., Pérez-Gracia J.L., Han J.-Y., Molina J., Kim J.-H., Arvis C.D., Ahn M.-J. (2016). Pembrolizumab versus docetaxel for previously treated, PD-L1-positive, advanced non-small-cell lung cancer (KEYNOTE-010): A randomised controlled trial. Lancet Lond. Engl..

[11] Michot J., Bigenwald C., Champiat S., Collins M., Carbonnel F., Postel-Vinay S., Berdelou A., Varga A., Bahleda R., Hollebecque A. (2016). Immune-related adverse events with immune checkpoint blockade: A comprehensive review. Eur. J. Cancer Oxf. Engl..

[12] Postow M.A., Sidlow R., Hellmann M.D. (2018). Hellmann MD. Immune-Related Adverse Events Associated with Immune Checkpoint Blockade. N. Engl. J. Med..

[13] Arnaud-Coffin P., Maillet D., Gan H.K., Stelmes J.-J., You B., Dalle S., Péron J. (2019). A systematic review of adverse events in randomized trials assessing immune checkpoint inhibitors. Int. J. Cancer.

[14] Attia P., Phan G.Q., Maker A.V., Robinson M.R., Quezado M.M., Yang J.C., Sherry R.M., Topalian S.L., Kammula U.S., Royal R.E. (2005). Autoimmunity correlates with tumor regression in patients with metastatic melanoma treated with anti-cytotoxic T-lymphocyte antigen-4. J. Clin. Oncol. Off. J. Am. Soc. Clin. Oncol..

[15] Teulings H.E., Limpens J., Jansen S.N., Zwinderman A.H., Reitsma J.B., Spuls P.I., Luiten R.M. (2015). Vitiligo-like depigmentation in patients with stage III-IV melanoma receiving immunotherapy and its association with survival: A systematic review and meta-analysis. J. Clin. Oncol. Off. J. Am. Soc. Clin. Oncol..

[16] Grangeon M., Tomasini P., Chaleat S., Jeanson A., Souquet-Bressand M., Khobta N., Bermudez J., Trigui Y., Greillier L., Blanchon M. (2019). Association Between Immune-related Adverse Events and Efficacy of Immune Checkpoint Inhibitors in Non-small-cell Lung Cancer. Clin. Lung. Cancer.

[17] Rogado J., Sánchez-Torres J., Romero-Laorden N., Ballesteros A., Pacheco-Barcia V., Ramos-Leví A., Arranz R., Lorenzo A., Gullón P., Donnay O. (2019). Immune-related adverse events predict the therapeutic efficacy of anti-PD-1 antibodies in cancer patients. Eur. J. Cancer Oxf. Engl..

[18] Ricciuti B., Genova C., De Giglio A., Bassanelli M., Bello M.G.D., Metro G., Brambilla M., Baglivo S., Grossi F., Chiari R. (2019). Impact of immune-related adverse events on survival in patients with advanced non-small cell lung cancer treated with nivolumab: Long-term outcomes from a multi-institutional analysis. J. Cancer Res. Clin. Oncol..

[19] Verzoni E., Cartenì G., Cortesi E., Giannarelli D., De Giglio A., Sabbatini R., Buti S., Rossetti S., Cognetti F., on behalf of the Italian Nivolumab Renal Cell Cancer Early Access Program Group (2019). Real-world efficacy and safety of nivolumab in previously-treated metastatic renal cell carcinoma, and association between immune-related adverse events and survival: The Italian expanded access program. J. Immunother. Cancer.

[20] Eggermont A.M., Kicinski M., Blank C.U., Mandala M., Long G.V., Atkinson V., Khattak A., Carlino M.S., Sandhu S., Suciu S. (2020). Association Between Immune-Related Adverse Events and Recurrence-Free Survival Among Patients With Stage III Melanoma Randomized to Receive Pembrolizumab or Placebo: A Secondary Analysis of a Randomized Clinical Trial. JAMA Oncol..

[21] Berner F., Bomze D., Diem S., Ali O.H., Fässler M., Ring S., Niederer R., Ackermann C.J., Baumgaertner P., Pikor N. (2019). Association of Checkpoint Inhibitor-Induced Toxic Effects With Shared Cancer and Tissue Antigens in Non-Small Cell Lung Cancer. JAMA Oncol..

[22] Akamatsu H., Murakami E., Oyanagi J., Shibaki R., Kaki T., Takase E., Tanaka M., Harutani Y., Yamagata N., Okuda Y. (2020). Immune-Related Adverse Events by Immune Checkpoint Inhibitors Significantly Predict Durable Efficacy Even in Responders with Advanced Non-Small Cell Lung Cancer. Oncologist.

[23] Arbour K.C., Mezquita L., Long N., Rizvi H., Auclin E., Ni A., Martínez-Bernal G., Ferrara R., Lai W.V., Hendriks L.E.L. (2018). Impact of Baseline Steroids on Efficacy of Programmed Cell Death-1 and Programmed Death-Ligand 1 Blockade in Patients with Non-Small-Cell Lung Cancer. J. Clin. Oncol. Off. J. Am. Soc. Clin. Oncol..

[24] Garant A., Guilbault C., Ekmekjian T., Greenwald Z., Murgoi P., Vuong T. (2017). Concomitant use of corticosteroids and immune checkpoint inhibitors in patients with hematologic or solid neoplasms: A systematic review. Crit. Rev. Oncol. Hematol..

[25] Maillet D., Corbaux P., Stelmes J.-J., Dalle S., Locatelli-Sanchez M., Perier-Muzet M., Duruisseaux M., Kiakouama-Maleka L., Freyer G., Boespflug A. (2020). Association between immune-related adverse events and long-term survival outcomes in patients treated with immune checkpoint inhibitors. Eur. J. Cancer Oxf. Engl..

[26] Haratani K., Hayashi H., Nakagawa K. (2020). Association of immune-related adverse events with immune checkpoint inhibitor efficacy: Real or imaginary?. BMC Med..

[27] Kobayashi K., Suzuki K., Hiraide M., Aoyama T., Yokokawa T., Shikibu S., Hashimoto K., Iikura Y., Sato H., Sugiyama E. (2020). Association of Immune-Related Adverse Events with Pembrolizumab Efficacy in the Treatment of Advanced Urothelial Carcinoma. Oncology.

[28] Okada N., Kawazoe H., Takechi K., Matsudate Y., Utsunomiya R., Zamami Y., Goda M., Imanishi M., Chuma M., Hidaka N. (2019). Association between Immune-Related Adverse Events and Clinical Efficacy in Patients with Melanoma Treated With Nivolumab: A Multicenter Retrospective Study. Clin. Ther..

[29] Haratani K., Hayashi H., Chiba Y., Kudo K., Yonesaka K., Kato R., Kaneda H., Hasegawa Y., Tanaka K., Takeda M. (2018). Association of Immune-Related Adverse Events With Nivolumab Efficacy in Non-Small-Cell Lung Cancer. JAMA. Oncol..

[30] Yee C., Thompson J.A., Roche P., Byrd D.R., Lee P.P., Piepkorn M., Kenyon K., Davis M.M., Riddell R., Greenberg P.D. (2000). Melanocyte destruction after antigen-specific immunotherapy of melanoma: Direct evidence of t cell-mediated vitiligo. J. Exp. Med..

[31] Patel V., Elias R., Formella J., Schwartzman W., Christie A., Cai Q., Malladi V., Kapur P., Vazquez M., McKay R. (2020). Acute interstitial nephritis, a potential predictor of response to immune checkpoint inhibitors in renal cell carcinoma. J. Immunother. Cancer.

[32] Brahmer J.R., Lacchetti C., Schneider B.J., Atkins M.B., Brassil K.J., Caterino J.M., Chau I., Ernstoff M.S., Gardner J.M., Ginex P. (2018). Management of Immune-Related Adverse Events in Patients Treated With Immune Checkpoint Inhibitor Therapy: American Society of Clinical Oncology Clinical Practice Guideline. J. Clin. Oncol. Off. J. Am. Soc. Clin. Oncol..

[33] Haanen JB A.G., Carbonnel F., Robert C., Kerr K.M., Peters S., Larkin J., Jordan K. (2018). Management of toxicities from immunotherapy: ESMO Clinical Practice Guidelines for diagnosis, treatment and follow-up. Ann. Oncol. Off. J. Eur. Soc. Med. Oncol..

[34] Patil P., Jia X., Hobbs B., Pennell N. (2019). MA03.01 The Impact of Early Steroids on Clinical Outcomes in Patients with Advanced NSCLC Treated with Immune Checkpoint Inhibitors- A Cancerlinq Cohort. J Thorac. Oncol..

[35] Hegde P.S., Chen D.S. (2020). Top 10 Challenges in Cancer Immunotherapy. Immunity.

[36] Johnson D.B. (2018). Toxicities and outcomes: Do steroids matter?. Cancer.

[37] Petrelli F., Signorelli D., Ghidini M., Ghidini A., Pizzutilo E.G., Ruggieri L., Cabiddu M., Borgonovo K., Dognini G., Brighenti M. (2020). Association of Steroids use with Survival in Patients Treated with Immune Checkpoint Inhibitors: A Systematic Review and Meta-Analysis. Cancers.

[38] Song J.M., Behera T.R., Demski K., Yurco A., Patil P.D., Funchain P. (2020). Prognosis of patients developing immune-related adverse events with immune checkpoint inhibitors in melanoma influenced by the ability to resume therapy. J. Clin. Oncol..

[39] Motzer R.J., Escudier B., McDermott D.F., Frontera O.A., Melichar B., Powles T., Donskov F., Plimack E.R., Barthélémy P., Hammers H.J. (2020). Survival outcomes and independent response assessment with nivolumab plus ipilimumab versus sunitinib in patients with advanced renal cell carcinoma: 42-month follow-up of a randomized phase 3 clinical trial. J. Immunother. Cancer.

[40] Santini F.C., Rizvi H., Plodkowski A.J., Ni A., Lacouture M.E., Gambarin-Gelwan M., Wilkins O., Panora E., Halpenny D.F., Long N.M. (2018). Safety and Efficacy of Re-treating with Immunotherapy after Immune-Related Adverse Events in Patients with NSCLC. Cancer Immunol. Res..

[41] Wong F., Al Ibrahim B., Walsh J., Qumosani K. (2019). Infliximab-induced autoimmune hepatitis requiring liver transplantation. Clin. Case Rep..

[42] Kok B., Lester E.L.W., Lee W.M., Hanje A.J., Stravitz R.T., Girgis S., Patel V., Peck J.R., Esber C., for the United States Acute Liver Failure Study Group (2018). Acute Liver Failure from Tumor Necrosis Factor-α Antagonists: Report of Four Cases and Literature Review. Dig. Dis. Sci..

[43] Robert C., Marabelle A., Herrscher H., Caramella C., Rouby P., Fizazi K., Besse B. (2020). Immunotherapy discontinuation—How, and when? Data from melanoma as a paradigm. Nat. Rev. Clin. Oncol..

