# Supplementary material for: Investigating the Impact of Immune-Related Adverse Events, Glucocorticoid Use and Immunotherapy Interruption on Long-Term Survival Outcomes
Source: Cancers (Basel). 2021 May 14;13(10):2365. doi: 10.3390/cancers13102365 (PMC8156819; doi:10.3390/cancers13102365)
Supplement: Supplementary file 1 [file cancers-13-02365-s001.zip › cancers-1169127-supplementary.pdf]

# Supplementary Materials:

# Investigating the Impact of Immune-Related Adverse Events, Glucocorticoid Use and Immunotherapy Interruption on Long-Term Survival Outcomes

**Charline Lafayolle de la Bruyère [1,*], Pierre Jean Souquet [2,3,4], Stéphane Dalle [2,3,5], Pauline Corbaux [1], Amélie Boespflug [2,3,5], Michaël Duruisseaux [2,3,6], Lize Kiakouama-Maleka [2,3,7], Thibaut Reverdy [1], Madeleine Maugeais [1], Gulsum Sahin [3], Denis Maillet [1,2,3] and Julien Péron [1,2,3,8]**

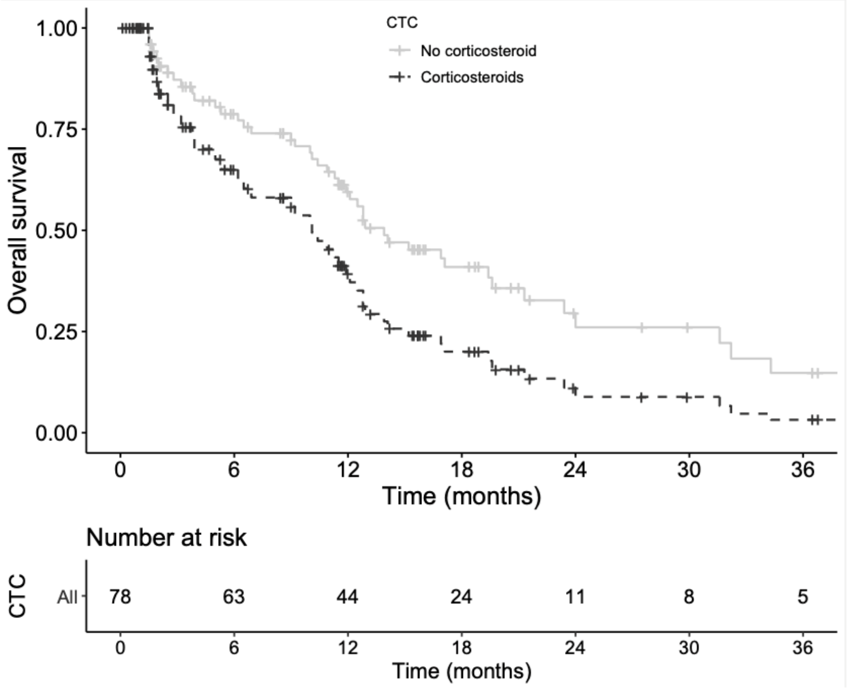


Figure S1 : Overall survival (OS) depending on the use of glucocorticoids or not in patients with grade 3 and more immune-related adverse events (irAEs).

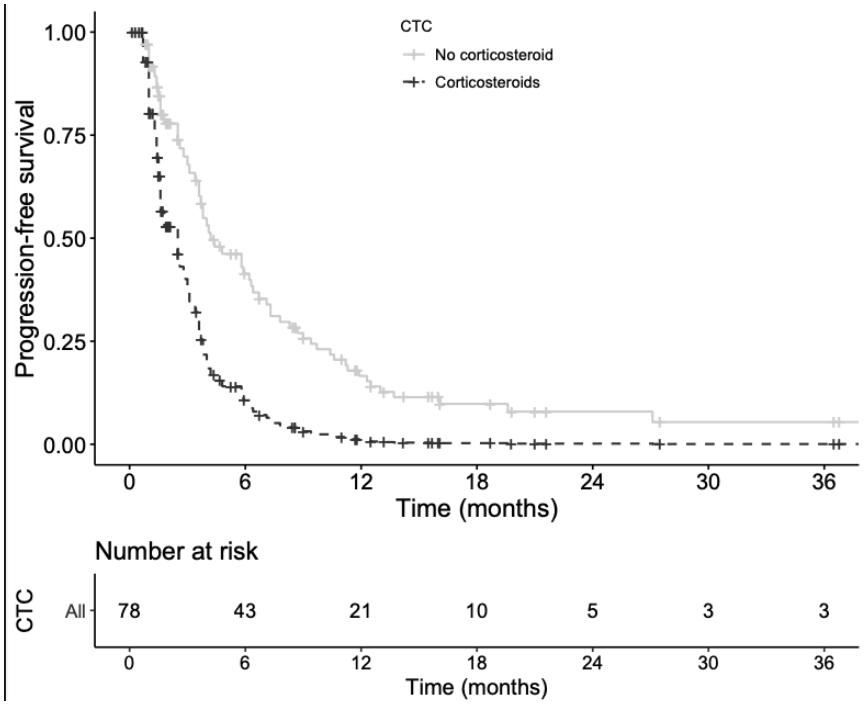


Figure S2 : Progression free survival (PFS) depending on the use of glucocorticoids or not in patients with grade 3 and more irAEs.

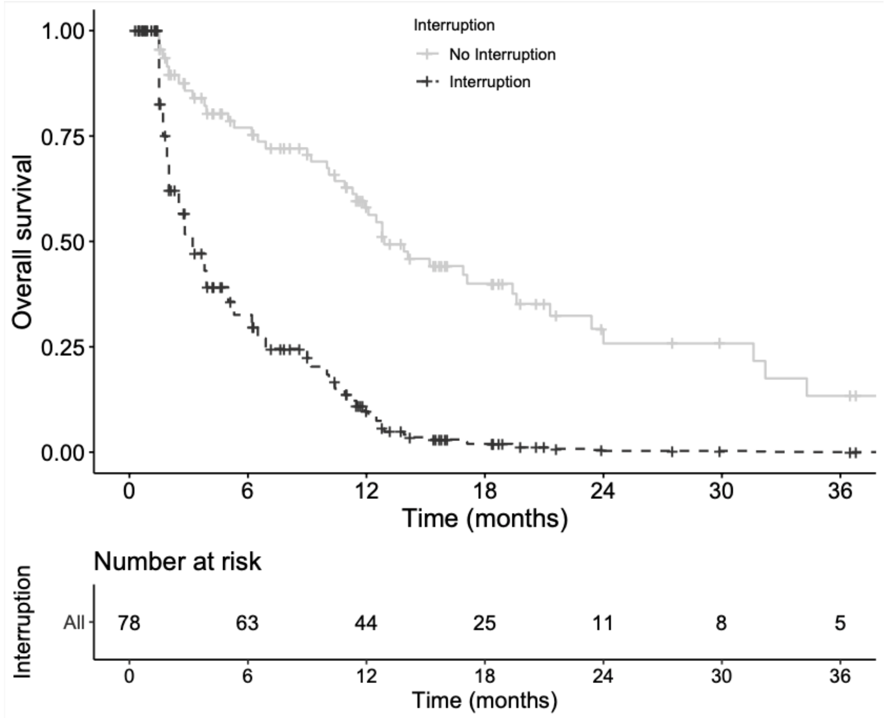


Figure S3 : OS depending on the interruption of immune check-point inhibitors or not in patients with grade 3 and more irAEs.

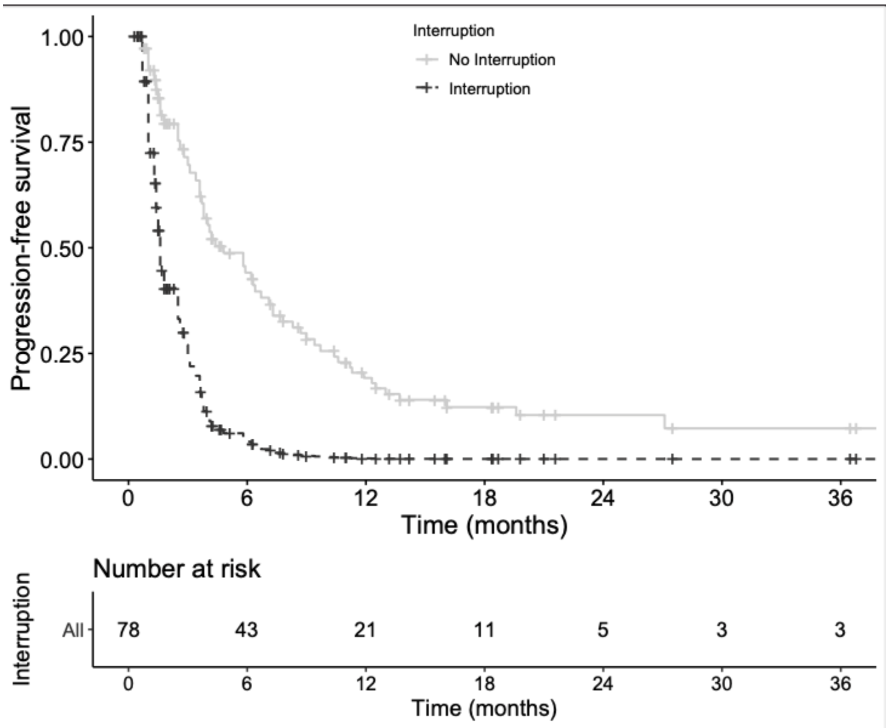


Figure S4 : PFS depending on the interruption of immune check-point inhibitors or not in patients with grade 3 and more irAEs.
